# Prognostic Nutritional Index and Neutrophil-to-Lymphocyte Ratio Are Respectively Associated with Prognosis of Gastric Cancer with Liver Metatasis Undergoing and without Hepatectomy

**DOI:** 10.1155/2019/4213623

**Published:** 2019-10-07

**Authors:** Jialiang Gao, Yimin Wang, Fengke Li, Ziyu Zhu, Bangling Han, Rui Wang, Rui Xie, Yingwei Xue

**Affiliations:** ^1^Department of Gastrointestinal Surgery, Harbin Medical University Cancer Hospital, 150 Haping Road, Nangang District, Harbin 150081, China; ^2^Department of Digestive Internal Medicine & Photodynamic Therapy Center, Harbin Medical University Cancer Hospital, 150 Haping Road, Nangang District, Harbin 150081, China

## Abstract

*Background. *To clarify the efficacy of hepatectomy for gastric cancer liver metastasis (GCLM) and to investigate the association between prognostic nutrition index (PNI) or neutrophil-to-lymphocyte ratio (NLR) and prognosis of GCLM undergoing or without hepatectomy. *Methods. *We retrospectively studied 374 patients with GCLM. The ROC curve was used to determine the optimal cut-off of PNI and NLR. Patients were divided into groups based on whether hepatectomy was performed, and survival analysis was conducted before and after grouping. The overall survival (OS) time and 1, 3, 5-year survival rates were also compared. *Results. *Multivariate analysis of all GCLM patients revealed that hepatectomy (*p* = 0.001) was an independent prognosis factor. And there were statistical differences in OS and 1, 3, 5-year survival rates (*p* = 0.001 of all) between hepatectomy group and nonhepatectomy group. Multivariate analysis of GCLM undergoing hepatectomy showed that PNI was an independent prognosis factor (*p* = 0.001). And there were statistical differences in OS and 1, 3, 5‐year survival rates (*p* = 0.001*p* = 0.005, *p* = 0.001 and *p* = 0.020, respectively) between high PNI group and low PNI group. Multivariate analysis of GCLM without hepatectomy showed that NLR was an independent prognosis factor (*p* = 0.001). And there were statistical differences in OS and 1, 3, 5-year survival rates (*p* = 0.001*p* = 0.008*p* = 0.031 and *p* = 0.026, respectively) between low NLR group and high NLR group. *Conclusions. *GCLM has a better prognosis with hepatectomy. High preoperative PNI is a benign prognostic predictor for patients undergoing hepatectomy. And high preoperative NLR is an adverse prognostic factor for patients without hepatectomy.

## 1. Introduction

Gastric cancer (GC) is a kind of malignant tumor with high morbidity and mortality [1]. Liver is one of the most common metastatic sites, and liver metastasis is a major factor leading to poor prognosis for GC [2, 3]. Hepatectomy, originally used to treat primary liver cancer or its recurrence, has been shown to be effective in treating liver metastases from digestive system [4, 5]. Whether hepatectomy can improve the prognosis of gastric cancer liver metastasis (GCLM) is still not fully determined [6, 7].

Prognosis and postoperative recovery of cancer patients are closely related to their nutritional status. Prognostic nutrition index (PNI) is calculated based on serum albumin level and total lymphocyte count in peripheral blood. It is initially used as a reflection of nutritional status for cancer patients [8]. It is shown that high PNI is a benign prognostic factor for multiple cancers [9–12]. However, the effect of PNI on the prognosis of GCLM still needs to be confirmed.

Neutrophil-to-lymphocyte ratio (NLR), as a common indicator of inflammation, has been used in the analysis of tumor-related inflammatory progression and prognosis. Patients with low NLR for breast cancer [13], liver cancer [14], colon cancer [15], and ovarian cancer [16], have a better prognosis. Although some studies [17] also have pointed out the association between NLR and prognosis of GC, NLR in the evaluation of prognosis of GCLM has not been found so far.

Thus, our present research was designed to clarify the efficacy of hepatectomy for GCLM. In addition, we aimed to investigate the association between PNI or NLR and the prognosis of GCLM patients undergoing or without hepatectomy.

## 2. Material and Method

### 2.1. Patients

A total of 475 GCLM patients, admitted to the Department of Abdominal or Gastrointestinal Surgery of Harbin Medical University Cancer Hospital from May 1975 to July 2013, were selected from the gastrointestinal surgery database. After expluding patients without any treatment (47 cases), who did not meet the blood test standards (14 cases), who did not test for albumin (PNI cannot be calculated, 40 cases), there were 374 patients remaining in this retrospective study.

### 2.2. Clinical Data Evaluation

The clinical data of all patients were complete. The diagnostic criteria and the evaluation for GCLM were in accordance in UICC&AJCC 8th edition. All the liver metastases of the patients were simultaneous in this study.

The patients' sex, age, the blood test results of blood type, the preoperative value of albumin, neutrophil count, and lymphocyte count were collected. The blood test results were obtained within 3 days before surgery, and the exclusion criteria for blood collection were as follows: (1) a history of exogenous blood transfusion for half a year, (2) new infection within half a month, (3) blood system diseases. The PNI was the sum of albumin value (g/L) and 5 times lymphocyte count (10^9^/L) [8]. The NLR value was the ratio of neutrophil count to lymphocyte count. Other clinicopathologic factors were also investigated, including T or N stage of GC tumors, gastrectomy and hepatectomy. And these potential prognostic factors are substituted into univariate analysis. Indications for hepatectomy include resection of the primary gastric tumor, and liver metastases are single or hemihepatic, and hepatectomy refers to partial hepatectomy or lobectomy involving liver metastases. In this study, all patients undergoing hepatectomy received radical gastrectomy, while those without hepatectomy only received palliative gastrectomy or laparotomy. All patients who underwent surgery did not undergo chemotherapy before surgery, and all patients receiving chemotherapy received at least one complete course of chemotherapy.

The patients were followed up for more than 3 months, at most 10 years. The deadline of follow-up was August 1, 2018. Overall survival (OS) time is defined as the duration of first diagnosis to patients died from GCLM or last follow-up.

### 2.3. Statistical Analysis

Age and survival time were represented by mean ± standard deviation (SD) and median (interquartile range), respectively. The continuous variables, analyzed by a *T* test, were utilized to select the optimal cut-off value using the receiver operating characteristic curve (ROC). The categorical variables were represented by percentages and evaluated using a χ^2^ test or Fisher's exact test.

A univariate survival analysis was performed using the Kaplan—Meier curve, and differences were evaluated according to the log-rank test. Variables proved to be statistically significant in the univariate analysis were included into the Cox's proportional hazard model for multivariable survival analysis, to identify the independent prognostic factors.

The experimental data were analyzed using SPSS19.0 statistical software (IBM, Chicago, IL, USA). A *p* value less than 0.05 was considered statistically significant.

## 3. Results

### 3.1. The Optimal Cut-Off of Clinical Indicators

**Figure 1:**
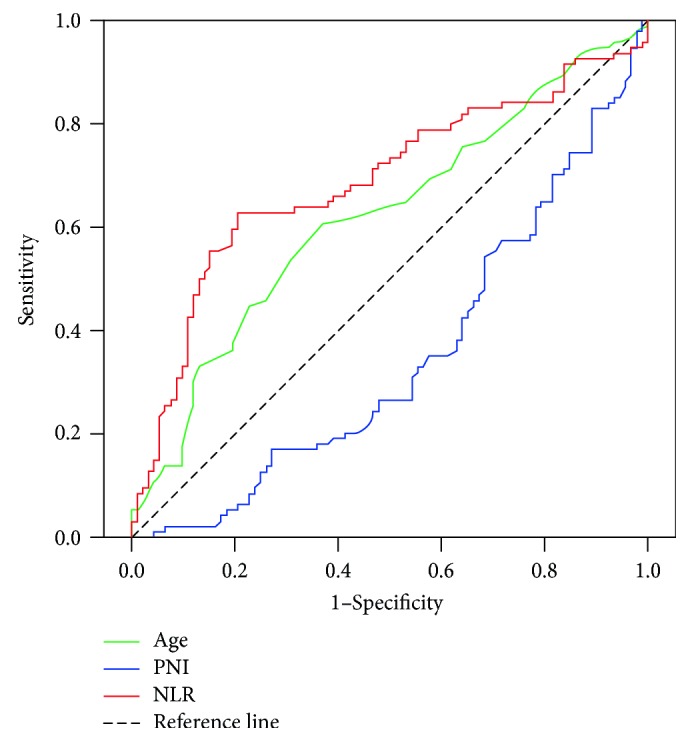
Survival ROC curve (with Youden index and *p* value) for age (0.236, *p* = 0.005), PNI (−0.198, *p* = 0.001) and NLR (0.421,*p* = 0.001).

In order to group and analyze the patients, ROC was used to seek the optimal cut-off of the clinical indicators, such as age, PNI and NLR. Based on the results, the age, PNI and NLR of GCLM patients are high when their value no less than 58, 46.8, and 2.86, otherwise they are low (Figure 1).

### 3.2. The Characteristics of All Patients and the Association between Hepatectomy and Their Prognosis

A total of 374 patients with GCLM were included in the study, including 299 (79.9%) males and 75 (20.1%) females. The median age was 56.8 ± 10.8 years old. The OS was 8.0 (16.0) months, with 1-year, 3‐year and 5‐year survival rates of 36.0%, 13.1%, and 6.7%, respectively. Hepatectomy was performed in 54 (14.4%) patients and not performed in 320 (85.6%) patients. (Table 1).

**Figure 2:**
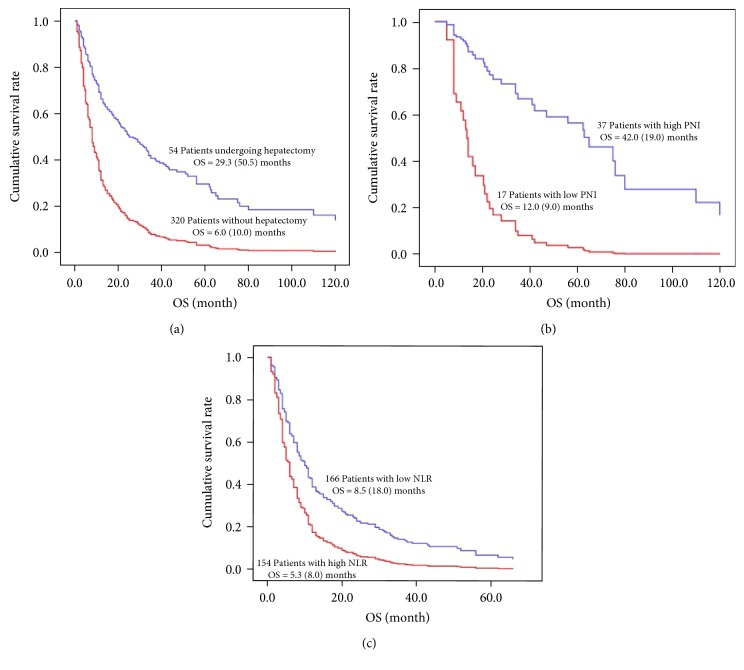
Kaplan-Meier survival curve. (a) For all patients, a significant difference was observed between patients undergoing hepatectomy and those without hepatectomy (*p* = 0.001). (b) For the patients undergoing hepatectomy. A significant difference was observed between high PNI group and low PNI group (*p* = 0.001). (c) For the patients without hepatectomy. A significant difference was observed between high NLR group and low NLR group (*p* = 0.001).

For all patients, univariate analysis demonstrated that the age, the N stage of tumor, the number of liver metastasis, NLR, PNI, gastrectomy, hepatectomy and chemotherapy exhibited significant differences in prognosis. And multivariate analysis revealed that hepatectomy and chemotherapy (*p* = 0.001 and *p* = 0.015) were independent prognosis factors (Table 1, Figure 2(a)).

The OS and 1, 3, 5-year survival rates of patients undergoing hepatectomy were better than the patients without hepatectomy (29.3 > 6.0 months, 77.8% > 29.0%, 37.0% > 9.0%, 25.9% > 3.4%, respectively). And there were statistical differences (*p* = 0.001 of all) between the two groups (Table 2).

### 3.3. The Association between PNI and Prognosis of the Patients Undergoing Hepatectomy

There were 54 patients undergoing hepatectomy, including 43 (79.6%) males and 11 (20.4%) females, with the median age was 57.0 ± 10.5 years old. Univariate and multivariate analysis showed that PNI (*p* = 0.001) was an independent prognosis factor for GCLM undergoing hepatectomy (Table 1, Figure 2(c)).

Meanwhile, the OS (42.0 > 12.0 months) and 1, 3, 5‐year survival rates (89.2% > 52.9%, 51.4% > 5.9%, 35.1% > 5.9%, respectively) of the 37 patients with high PNI were better than the 17 patients with low PNI, and the difference was statistically significant (*p* = 0.001, *p* = 0.005,*p* = 0.001, and *p* = 0.020, respectively) (Table 2).

### 3.4. The Association between NLR and Prognosis of the Patients without Hepatectomy

There were 320 patients without hepatectomy, including 256 (80.0%) males and 64 (20.0%) females, with the median age was 56.8 ± 10.9 years old. Univariate and multivariate analysis showed that NLR (*p* = 0.001) and chemotherapy (*p* = 0.021) were independent prognosis factors for GCLM without hepatectomy. (Table 1, Figure 2(c)).

Meanwhile, The OS and 1, 3, 5-year survival rates of the 166 patients with low NLR were better than the 154 patients with high NLR (8.5 >  5.3  months, 37.2% >  22.7%, 13.3% >  4.5%, 5.4% > 1.3%, respectively). And there were statistical differences in all of them (*p* = 0.001, *p* = 0.008, *p* = 0.031, and *p* = 0.026, respectively) between the two groups. (Table 2).

### 3.5. The Comparison of Characteristics between GCLM Undergoing and without Hepatectomy

There was no significant difference in their characteristics including gender (*p* = 0.538), age (*p* = 0.206), the count of albumin (*p* = 0.674), lymphocyte (*p* = 0.206) and neutrophil (*p* = 0.540), the N stage of the gastric tumor (*p* = 0.499), chemotherapy (*p* = 0.451), PNI (*p* = 0.907) and NLR (*p* = 0.936), except for more the number of T4b (62.8% > 46.3%, *p* = 0.017) and liver metastases (70.4% >  37.5%, *p* = 0.001) and less gastrectomy (32.8% < 100.0%, *p* = 0.001) in nonhepatectomy group (Table 3). It may be due to the large extent of resection required for radical surgery, the inability to reconstruct the digestive tract after surgery or the inability to ensure residual organ function.

## 4. Discussion

**Figure 3:**
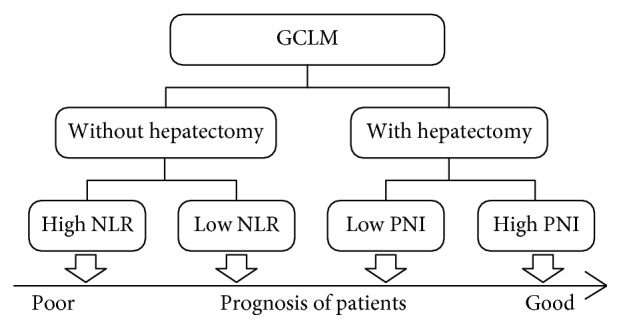
Prognostic analysis of GCLM obtained from our study, patients were classified by hepatectomy at first, then by PNI or NLR.

After a retrospective analysis of 374 patients with GCLM, we found that patients undergoing hepatectomy had good prognosis, and among them, those with high PNI before hepatectomy had better prognosis. However, the patients without hepatectomy had poor prognosis, and among them, those with low NLR had worse prognosis (Figure 3).

Current studies on surgical treatment of GCLM are mainly retrospective studies from a single center. GCLM case samples are small, patients' survival time and long-term survival rate are still low after treatment, and long-term survival has been observed only in a few selected cases [18–20]. Therefore, we collected more patients in this study. Multivariate analysis showed that hepatectomy could significantly improve patients' prognosis and survival (*p* = 0.001). The median OS (29.3** **months) and 1-year (77.8%), 3-year (37.0%) and 5-year (25.9%) survival rates of the patients undergoing hepatectomy were significantly superior to those of GCLM patients without hepatectomy. These results suggested that the hepatectomy may be effective. A similar research on the short-term safety and long-term survival benefits of GCLM resection, from England in 2016, also confirmed that hepatectomy has some efficacy in survival [21].

It is well-known that nutrition plays a crucial part in immune system. Malnutrition inhibits innate and cellular immunity, which in turn makes the body vulnerable to infection and cancer [22, 23]. Several studies from both east and west have pointed out that preoperative low PNI was associated with poor prognosis of patients with GC [9], liver tumor [10], colorectal cancer liver metastasis [11] and malignant tumor after radical surgery [12]. In our study, PNI was still an independent prognosis factor for GCLM undergoing hepatectomy (*p* = 0.001). Decreased PNI is caused by lymphocyte depletion and hypoalbuminemia. Lymphocytes kill off new cancer cells, and a low lymphocyte count may reflect a lack of tumor immunity [24, 25]. There's a lot of inflammatory or pro-inflammatory cytokines, including interleukin-1, interleukin-6, and tumor necrosis factor alpha (TNF-α), produced in the chronic inflammatory response. They can cause hypoalbuminemia and lead to cancer [26–28]. It has been reported that lymphocyte reduction and hypoalbuminemia are independent prognostic factors for colorectal and renal cell cancers [29, 30]. Therefore, PNI as the sum of the above two may have similar or even better prognostic value for GCLM. Finally, as a prognostic predictor, PNI has the advantage of being convenient to detect.

NLR is an indicator of systemic inflammation, and the higher the NLR, the greater the inflammatory response. There have been many reviews and meta-analyses showing that NLR is a prognostic factor for various cancers [13–16]. Our multivariate analysis demonstrated that NLR was an independent prognosis factor for GCLM without hepatectomy (*p* = 0.001). Patients with high preoperative NLR had worse OS and 1, 3, 5-year survival rates. High NLR is associated with systemic inflammatory response. And the inflammation enhances the cascade of events associated with inflammatory cytokines dominated by TNF-α and interleukins. This may be the mechanism by which high NLR results in poor cancer prognosis. These immunomodulators can affect the function of natural killer cells and cytotoxic T lymphocytes, and increase the accumulation of tumor-associated macrophages. In turn, micrometastasis develops rapidly during the period of weak immunity due to postoperative complications [31–33].

Our survival analysis found that for general GCLM, palliative chemotherapy can improve the prognosis (*p* = 0.015), as well as for GCLM without hepatectomy (*p* = 0.021), and may also have a certain effect on the hepatectomy group (*p* = 0.167). Undoubtedly, palliative chemotherapy can improve the prognosis of GCLM. However, chemotherapy was an independent prognostic factor in this study and was independent of whether or not hepatectomy was performed. There was no difference in the amount of chemotherapy received between the two groups of GCLM (*p* = 0.451).

In conclusion, GCLM has a better prognosis with hepatectomy. For patients undergoing hepatectomy, PNI is an effective prognostic predictor. Patients with high preoperative PNI have good prognosis and lasting survival. For patients without hepatectomy, NLR is a more appropriate prognostic factor, and patients with high preoperative NLR have poor prognosis and few survival.

## Figures and Tables

**Table 1:** Characteristics and survival analysis of all patients of GCLM.

Patients	All GCLM			GCLM undergoing hepatectomy			GCLM without hepatectomy		
Parameter	*n* = 374*n* (%)	*p*		*n* = 54*n* (%)	*p*		*n* = 320*n* (%)	*p*	
		Univariate	Multivariate		Univariate	Multivariate		Univariate	Multivariate
Gender		0.409	0.258		0.078	0.111		0.477	0.254
Male	299 (79.9)			43 (79.6)			256 (80.0)		
Female	75 (20.1)			11 (20.4)			64 (20.0)		
Age (57 ± 11)		0.038^∗^	0.404		0.341	0.722		0.151	0.489
<58	191 (51.1)			29 (53.7)			162 (50.6)		
≥58	183 (48.9)			25 (46.3)			158 (49.4)		
T stage		0.144	0.796		0.417	0.067		0.724	0.972
T4b	226 (60.4)			25 (46.3)			201 (63.6)		
Not T4b	148 (39.6)			29 (53.7)			119 (36.4)		
N stage		0.027^∗^	0.508		0.234	0.481		0.389	0.770
N0 or N1	122 (32.6)			18 (33.3)			104 (32.5)		
N2 or N3	252 (67.4)			36 (66.7)			216 (67.5)		
Number of liver metastases		0.048^∗^	0.560		0.824	0.444		0.562	0.433
Single	158 (42.2)			38 (70.4)			120 (37.5)		
Multiple	215 (57.8)			16 (29.6)			200 (62.5)		
Gastrectomy		0.001^∗^	0.059		‐‐‐	‐‐‐		0.037^∗^	0.096
Yes	159 (42.5)			54 (100.0)			105 (36.0)		
No	215 (57.5)			0 (0.0)			215 (64.0)		
Hepatectomy		0.001^∗^	0.001^∗^						
Yes	54 (14.4)								
No	320 (85.6)								
Chemotherapy		0.016^∗^	0.015^∗^		0.278	0.167		0.011^∗^	0.021^∗^
Yes	160 (42.8)			24 (44.4)			136 (42.5)		
No or Unknown	214 (57.2)			30 (55.6)			184 (57.5)		
PNI		0.001^∗^	0.113		0.001^∗^	0.001^∗^		0.307	0.871
<46.8	140 (37.4)			17 (31.5)			123 (38.4)		
≥46.8	234 (62.6)			37 (68.5)			197 (61.6)		
NLR		0.001^∗^	0.099		0.127	0.733		0.001^∗^	0.001^∗^
<2.86	201 (53.7)			35 (64.8)			166 (49.6)		
≥2.86	173 (46.3)			19 (35.2)			154 (50.4)		

^∗^ *p* < 0.05.

**Table 2:** Comparison of survival in operation for all patients of GCLM.

Parameter		All	Yes/High	No/Low	*p*
Hepatectomy in all GCLM, Yes : No = 54 : 320	OS (month)	8.0 (16.0)	29.3 (50.5)	6.0 (10.0)	0.001^∗^
	1‐year survival, *n* (%)	135 (36.0%)	42 (77.8%)	93 (29.0%)	0.001^∗^
	3-year survival, *n* (%)	49 (13.1%)	20 (37.0%)	29 (9.0%)	0.001^∗^
	5‐year survival, *n* (%)	25 (6.7%)	14 (25.9%)	11 (3.4%)	0.001^∗^
PNI in GCLM undergoing hepatectomy, High : Low = 37 : 17	OS (month)	29.3 (50.5)	42.0 (49.0)	12.0 (9.0)	0.001^∗^
	1‐year survival, *n* (%)	42 (77.8%)	33 (89.2%)	9 (52.9%)	0.005^∗^
	3‐year survival, *n* (%)	20 (37.0%)	19 (51.4%)	1 (5.9%)	0.001^∗^
	5‐year survival, *n* (%)	14 (25.9%)	13 (35.1%)	1 (5.9%)	0.020^∗^
NLR in GCLM without hepatectomy, High : Low = 154 : 166	OS (month)	6.0 (10.0)	5.3 (8.0)	8.5 (18.0)	0.001^∗^
	1‐year survival, *n* (%)	93 (29.0%)	35 (22.7%)	58 (37.2%)	0.008^∗^
	3-year survival, *n* (%)	29 (9.0%)	7 (4.5%)	22 (13.3%)	0.031^∗^
	5-year survival, *n* (%)	11 (3.4%)	2 (1.3%)	9 (5.4%)	0.026^∗^

^∗^ *p* < 0.05.

**Table 3:** Comparison of characteristics between GCLM undergoing and without hepatectomy.

Patients	GCLM undergoing hepatectomy, *n* = 54	GCLM without hepatectomy, *n* = 320	*p*
Parameter	*n* (%)/mean ± SD		
Gender			0.538
Male	43 (79.6)	256 (80.0)	
Female	11 (20.4)	64 (20.0)	
			
Age	57.0 ± 10.5	56.8 ± 10.9	0.206
Albumin (g/L)	39.3 ± 5.10	39.7 ± 5.70	0.674
Lymphocyte (/10^9^L)	2.50 ± 1.56	2.25 ± 1.34	0.206
Neutrophil (/10^9^L)	4.63 ± 2.75	4.86 ± 2.48	0.540
T stage			0.017^∗^
T4b	25 (46.3)	201 (62.8)	
Not T4b	29 (53.7)	119 (37.2)	
N stage			0.499
N0 or N1	18 (33.3)	104 (32.5)	
N2 or N3	36 (66.7)	216 (67.5)	
Number of liver metastases			0.001^∗^
Single	38 (70.4)	120 (37.5)	
Multiple	16 (29.6)	200 (62.5)	
Gastrectomy			0.001^∗^
Yes	54 (100.0)	105 (32.8)	
No	0 (0.0)	215 (67.2)	
Chemotherapy			0.451
Yes	24 (44.4)	136 (42.5)	
No or Uncertain	30 (55.6)	184 (57.5)	
PNI	49.0 ± 6.70	49.0 ± 7.17	0.907
NLR	3.35 ± 2.86	3.32 ± 2.11	0.936

^∗^ *p* < 0.05.

## Data Availability

The datasets generated and analysed during the current study are not publicly available. Because the data came from the hospital's independent and closed digital medical record management system. But they are available from the corresponding author on reasonable request.
